# China’s Insurance Regulatory Reform, Corporate Governance Behavior and Insurers’ Governance Effectiveness

**DOI:** 10.3390/ijerph14101238

**Published:** 2017-10-17

**Authors:** Huicong Li, Hongliang Zhang, Sang-Bing Tsai, Aichao Qiu

**Affiliations:** 1 Business School of Beijing Technology and Business University, Beijing 100048, China; lihuicong@btbu.edu.cn; 2 Zhongshan Institute, University of Electronic Science and Technology of China, Zhongshan 528400, China; 3 Economics and Management College, Civil Aviation University of China, Tianjin 300300, China; 4 Yingda International Trust Co. Ltd., Beijing 100005, China; yzxiaoai6416@163.com

**Keywords:** corporate governance regulation, corporate governance effectiveness, green governance, green operation

## Abstract

External regulation is an important mechanism to improve corporate behavior in emerging markets. China’s insurance governance regulation, which began to supervise and guide insurance corporate governance behavior in 2006, has experienced a complex process of reform. This study tested our hypotheses with a sample of 85 firms during 2010–2011, which was obtained by providing a questionnaire to all of China’s shareholding insurance companies. The empirical study results generally show that China’s insurance governance effectiveness has significantly improved through strict regulation. Insurance corporate governance can improve business acumen and risk-control ability, but no significant evidence was found to prove its influence on profitability, as a result of focusing less attention on governance than on management. State ownership is associated with higher corporate governance effectiveness than non-state ownership. Listed companies tend to outperform non-listed firms, and life insurance corporate governance is more effective than that of property insurers. This study not only contributes to the comprehensive understanding of corporate governance effectiveness but also to the literature by highlighting the effect of corporate governance regulation in China’s insurance industry and other emerging economies of the financial sector.

## 1. Introduction

External regulation is an important mechanism for improving corporate behavior, especially in emerging markets where the firm’s behavior needs to be improved (LLSV, 1998 [1]). Governments have noted the importance of financial institution governance after the 2008 financial crisis, and introduced a series of policies to regulate the financial industry given their negative externality. China’s insurance industry has become one of the fastest growing sectors in the national economy, playing a significant role in financial intermediation and risk-taking, second only to banking. The China Insurance Regulatory Commission (CIRC) introduced the ‘Guiding Opinions on Regulating the Insurance Corporate Governance Structure (Trial)’ in 2006 to supervise and guide insurance corporate governance using the core principles of International Association of Insurance Supervisors (IAIS) for reference. Furthermore, the CIRC has established three pillars of regulation—market behavior, corporate governance, and solvency—as a landmark beginning of insurance corporate governance reform. After more than a decade of development, Chinese insurance companies have formed a new pattern, encouraging fair competition and normal development (Chinese Academy of Social Sciences Institute of Finance, 2012). Chinese joint-stock insurance companies, such as People’s Insurance Company of China and China Life, are constantly improving their governance structures and operation mechanisms. They have gradually built insurance corporate governance structures and mechanisms with Chinese characteristics, based on the international experience and the realities of China’s insurance industry. Behind these regulatory compliance actions, many unsatisfactory cases still exist. The Chinese regulatory authority has increased the speed with which insurance licenses are issued. From October 2011 to April 2017, more than 50 licenses were issued and nearly 200 insurance companies were preparing to apply for a license. The ‘barbaric growth’ of the insurance industry creates a series of problems for the corporate governance of insurance companies. Large enterprise groups control an insurance company through a complex holding relationship, making it an internal cash dispenser, which hurts the interests and the trust of the insured and society. Chinese insurance companies are focused on establishing a modern enterprise system and governance structure according to the regulations. Even when all insurance companies have established an appropriate governance structure, the effects have been proven to be completely disparate, where many companies were able to operate continuously, while others collapsed rapidly. China’s insurance corporate governance has improved since the comprehensive governance was introduced 2006, but governance failures still abound, uncovering the limited effectiveness of the dominant governance structures and mechanisms.

This study focuses on three main research questions: (1) What is the effectiveness of corporate governance in the insurance industry, and how can it be measured? (2) What is the effect of China’s governance of insurance companies? (3) What are the implications of the nature of equity and does listing affects the effectiveness of corporate governance? To address these questions, this paper aimed to summarize the role of corporate governance in transition economies like China. This paper defines corporate governance effectiveness under the framework of new institutional economics along with governance structure and mechanisms required to be effective. Considering the complexity of financial institutions and insurers, this paper, deepening the study of insurance corporate governance, proposes that the effectiveness of corporate governance is manifest in three aspects: business, profitability, and risk control. This paper compared various measures on efficiency and chose a stochastic distance function approach, applicable to financial institutions in transition economies, to measure the effectiveness of corporate governance. This choice is consistent with our concept definition and extends the applicable scope of the classical method, so that the conclusion could be applied to understand and improve the effectiveness of corporate governance in other countries and industries.

This study tested our hypotheses by using a sample of questionnaires from 85 Chinese joint-stock insurance companies obtained from the full sample completed through regulatory authorities. The paper provides an up-to-date assessment of Chinese insurance corporate governance to supplement the lack of empirical research. As of October 2014, there are 64 property insurance companies, 76 life insurance companies, 18 insurance asset management companies, as well as 8 reinsurers. Although many studies have been completed on insurer corporate governance (Barrese et al., 2007 [2]) and on the efficiency of other financial institutions (Yao et al., 2007 [3]), few studies or little empirical research can be found on China’s insurance corporate governance. This is partly due to the lack of information, with only five companies listed on the stock exchange.

Our essay is organized as follows. The next section briefly reviews the evolution of China’s insurance industry reform, as well as literature on insurance corporate governance and its effectiveness. Section 3 describes research methodologies. Section 4 specifies an empirical model and describes the data. Our initial empirical analysis is presented in Section 5. Section 6 draws conclusions.

## 2. Theoretical Background

### 2.1. Insurers’ Governance Effectiveness

Previous studies have confirmed that reasonable governance structures and mechanisms can significantly enhance corporate value (Chhaochharia and Grinstein, 2007 [4]). Cao and Qian (2011) proposed that the effects of corporate governance include improvement of corporate performance, convenience benefits, such as reducing financing costs and enhancing market reputation, wise decision-making, and the reduction of agency costs [5]. Therefore, the most common definition of corporate governance effectiveness is from the perspective of business performance, as the impacts on increasing profit and the value of corporate governance mechanisms are based on the shareholder primacy theory (Lin and Zhang, 2009 [6]; Conheady et al., 2014 [7]). 

In addition, studies have shown that the role of corporate governance in risk control for investment, mergers, and acquisitions has become more obvious (Cheng et al., 2011 [8]). As a system of protection, corporate governance is conducive to ensuring the insurers operate steadily. The literature on the governance of insurance companies and other financial institutions have investigated the association between corporate governance and risk control. Therefore, the effectiveness of insurer governance should also include a reduction in governance risks and compliance risks as a result of its improvement.

### 2.2. Measurement of the Insurers’ Governance Effectiveness

The measurement of governance effectiveness has been extensively covered in the literature; however, there is no consensus on how to measure it. 

Numerous studies have used market value (Barnhart and Rosensteince, 1998 [9]) and financial performance (Mercedes, 2016 [10]) as standard evaluations of corporate governance effectiveness. From a stakeholder theory perspective, the literature ranges from profit to operating stability for protecting stakeholders (Brick and Chidambaran, 2008 [11]). Many evaluation standards about risk are used to investigate this concept, such as the Z index (Laeven and Levine, 2009 [12]) and market beta (Elyasian and Zhang, 2015 [13]). This perspective confuses business performance with corporate governance effectiveness. Although effective corporate governance is associated with increased business performance, this does not mean that all revenue comes from corporate governance or increases in performance are solely due to corporate governance. Especially because of China’s immature capital market, market value and financial performance indicators are more susceptible to tampering, so they may not effectively reflect the actual governance effect.

Another area of corporate governance effectiveness is minimizing governance costs (Benson et al., 2011 [14]). According to principal-agent theory, the narrow concept of governance cost is limited to the agency costs, including monitoring costs by the principal, bonding costs by the agent, and residual loss (Jensen and Meckling, 1976 [15]). Since then, governance costs have been further broadened to include agency and transaction costs. Hart and Moore (1996) considered that an effective corporate governance structure should be able to reduce agency and transaction costs within accompany [16]. Li and Wu (1999) defined corporate governance costs as the costs for maintaining governance structures and the required mechanisms [17]. As seen from the change of definition, many scholars regard corporate governance effectiveness as institutional arrangements to reduce the cost of governance. Such a perspective simplifies corporate governance effectiveness as reducing costs, which increases the research operability but ignores the benefits of governance.

To fully investigate the effectiveness of corporate governance, some scholars considered the benefits and costs of governance. This view regards corporate governance effectiveness as maximizing benefits and minimizing costs of governance. Because defining governance benefits and costs is difficult, the literature has not reached a consensus about how to measure relative benefits and costs.

Frontier analysis is an improvement on the relative view. With efficiency analysis, the resources usually refer to inputs and the outcomes refer to outputs. Frontier efficiency measures the ratio of an observed output over the maximum possible output, or the frontier input level, by the given technical and external environment at a particular point in time. Frontier analysis is more prevalent in insurers’ studies. Early empirical papers adopted a two-stage approach to investigate the influence of corporate governance by calculating technical efficiency using data envelope analysis (DEA) or the stochastic frontier analysis (SFA) approach in the first stage. The second stage involved a regression model for the technical inefficiency effects of corporate governance (Huang et al., 2011 [18]). Kumbhakar (1991) [19], and Battese and Coelli (1995) proposed models for technical inefficiency effects involved in frontier function [20]. The parameters of the frontier and the inefficiency model are estimated simultaneously, given appropriate distributional assumptions. This approach provides great convenience for studying the impacts of corporate governance on cost and profit efficiency.

### 2.3. Enhancing the Effectiveness of Insurers’ Governance

Both the theories of government regulation and institutional change discuss the effect of corporate governance, believing that improving corporate governance could mitigate principal-agent issues and reduce agency costs.

According to the theory of government regulation, the government regulates to maintain social welfare fairness as a neutral party when personal and social welfare conflict. Insurance companies have a wide range of stakeholders, so governments and regulators need to act as representatives of core stakeholders to implement regulation. The government’s fundamental purpose is to ensure that the corporate governance structure and mechanisms are established at a certain level in order to reduce risk and to protect the interests of the majority of stakeholders and social stability from the perspective of the overall interest of the entire society (Xia and Jin, 2013 [21]; Boot and Schmeits, 2000 [22]; Nier and Baumann, 2006 [23]).

According to the theory of institutional change and innovation, compliance is the action response that enterprises use to weigh regulatory requirements with their own interests. Therefore, enterprises may be non-compliant, or further improve their governance above the minimum regulatory requirements, in order to achieve internal organizational innovation based on the guidance of the regulatory authorities. Institutional change theory holds that institutional improvement can reduce uncertainties and the likelihood of opportunistic behavior. In practice, enterprises can obtain direct benefits from the capital market due to improved governance, and gain indirect benefits from the internal complex proxy relationship and cutting the costs of agency.

## 3. Concepts and Dimensions of Insurers’ Governance Effectiveness

### 3.1. Definition and Hypothesis of Insurers Governance Effectiveness: Based on the Perspective of Efficiency

#### 3.1.1. Effect of Corporate Governance

Agency theory states that corporate governance is concerned with the way in which owners monitor and control managerial performance to achieve their wealth maximization objectives (Nelson, 2005 [24]). With a feasible corporate governance system, the owners can be assured that managers use shareholders’ capital efficiently, thus receiving a competitive return on their investment (Shleifer and Vishny, 1997 [25]). Li (2007) proposed that corporate governance is responsible for integrating all resources to maintain a sustainable competitive advantage [26]. This paper argues that, unlike input factors such as labor, capital, and material resources, corporate governance can help use inputs more effectively to produce outputs by affecting input costs and resource allocation efficiency.

In terms of the impact on input costs, corporate governance can attract capital, labor, and other production factors by ensuring the interests of investors and employees. According to the study by McKinsey & Company (2002) [27], investors are willing to pay higher prices for a company’s corporate governance, which proves that good corporate governance can attract capital. Banks and financial institutions often consider the company’s governance when issuing loans to evaluate the capital security. Therefore, excellent corporate governance can reduce financing costs.

In terms of the impact on resource allocation efficiency, studies have found that corporate governance could improve the internal resource allocation efficiency. The primary objective of corporate governance is to coordinate all stakeholders and achieve sound decision-making. Therefore, corporate governance is conducive to improving the allocation efficiency of input factors by preventing investment failures and managers’ self-interested behavior.

#### 3.1.2. Definition of the Effectiveness of Insurers’ Governance

The new institutional economics theory suggests that institutions and institutional innovation significantly influence performance (Williamson, 1979 [28]). An enterprises’ efficiency is not only affected by cost and technology but also property and institutions themselves. Therefore, the study of performance efficiency, relying on effective institutional arrangements, needs to consider institutional factors. Corporate governance can affect productivity and improve performance as an environmental factor by attracting investments, reducing costs, and improving resource allocation efficiency. This paper defines corporate governance effectiveness as the efficiency improvement effected by corporate governance based on the new institutional economics theory.

China’s practices are a good proof of this effect. The insurance industry in China has developed considerably since joining the World Trade Organization (WTO). China’s insurance companies have improved their governance structure and standardized the responsibilities of shareholders, the board of directors, the board of supervisors, and executives. They transferred the policy-driven governance system to a market-oriented system and have achieved a balance in governance mechanisms amongst the authorities, decision-makers, supervisors, and managers. A series of reforms have significantly reduced the governance risks in China’s insurance industries, according to the China academy of Corporate Governance of Nankai University report (2014, 2017) [29,30]. Therefore, this study considers that the effectiveness of insurers’ governance is reflected in the reduction in governance and compliance risks.

As China’s market economy develops, insurance companies could impress investors in the capital market because of their improvement in corporate governance since 2016, individual insurance companies have encountered governance crises and are controlled by the consortium for irrational investment, resulting in significant losses. Enterprises with better governance can effectively avoid blind decision-making by using a rational decision-making mechanism, which not only guarantees business benefits, but also increases the trust of investors.

Based on the above analysis, this study suggests that the effectiveness of insurer governance consists of risk control, which is the effectiveness of risk reduction, as well as business and profitability effectiveness, which aim to enhance performance, according to stated business objectives and insurers’ governance specifics.

#### 3.1.3. Risk Control Effectiveness

Due to the governance and operational intricacies of insurance companies—including high debt ratio, dispersion of creditors, long-term insurance contracts, and specialization of the insurance product—insurance companies should consider not only maximizing shareholder value but also their ability to control risk. Existing studies have found that the effect of corporate governance on risk control is obvious. Ling et al., (2013) studied the U.S. property insurance companies, confirming that increasing the proportion of independent members on the board of directors can reduce both the investment risk and total risk [31]. Cheng et al., (2011) found that, as large shareholders, institutional investors can control investment risks and reduce the cost of capital [8]. The Chinese consortiums have acquired insurance companies and have treated them as their own sources of funds by intervening in the operation of the insurance companies and triggering industry risks. In this situation, the companies with good corporate governance can effectively resist foreign takeovers, thereby avoiding risks, and the companies with weaker corporate governance are likely to fall victim to this crisis. Corporate governance is indeed an effective barrier for insurance companies to control risk. Based on this, this paper presents the first hypothesis:

**Hypothesis** **1:** Improving insurance corporate governance is helpful for improving risk control efficiency.

#### 3.1.4. Business Effectiveness

The focus of Chinese insurance companies is on business and market share expansion and the success or failure is determined by premiums. Thus, research on the influence of corporate governance in business and management is of particular significance and interest. Previous research has documented that a sound system of governance could help insurers realize operational efficiencies and enhance economic performance (Huang et al., 2011 [18]). Its impact includes reducing agency costs (Wang et al., 2007 [32]), distributing power among stakeholders, avoiding manager entrenchment behavior (Raheja, 2005 [33]), and restructuring business initiatives (Thompson and Wright, 1995 [34]). Based on this, this paper presents the second hypothesis:

**Hypothesis** **2:** *Improving insurance corporate governance is helpful for improving business efficiency.*

#### 3.1.5. Profitability Effectiveness

Chinese insurance companies also need to improve their own profitability. The direction of the reform has been from extensive development to intensive development. Numerous studies have found that a high-quality governance structure can effectively avoid inefficient investment to prevent financial troubles (Li et al., 2011 [35]). The China Corporate Governance Evaluation Report reflects that good corporate governance will enable a company to maintain higher financial security in the future, thus helping improve the company’s profitability (China Academic of Corporate Governance of Nankai University). Diacon and O’Sullivan (1995) studied the impact of a board governance mechanism on a U.K. insurance company’s performance, and found that the professional committee had a greater influence on curbing executive pay, exerting a positive impact on company earnings [36]. Zhu and Wang (2017) have validated this effect in China’s insurance industry [37]. Their study found that insurance corporate governance can significantly reduce the probability of investment failure, and increase investment income. Based on this, this paper presents the third hypothesis:

**Hypothesis** **3:** Improving insurance corporate governance is helpful for improving profitability efficiency.

### 3.2. Measurementof Insurers’ Governance Effectiveness

#### 3.2.1. Measurement of Corporate Governance Inputs

This paper used the corporate governance inputs to evaluate the overall effectiveness of corporate governance. Various governance mechanisms intersect to achieve a governance effect (Cai and Wu, 2006 [38]), thereby maximizing the linear combination of governance indicators by factor analysis. Xiao (2005) mitigated the measurement error by using the same method as in the empirical analysis of the impact on capital structure of corporate governance [39]. Various corporate governance mechanisms interact with each other and combine to affect a company’s performance. The study also chose factor analysis, incorporating a set of corporate governance mechanisms into one indicator. 

#### 3.2.2. Measurement of the Effectiveness of Insurers’ Governance

‘Efficiency’ can be defined in different ways, such as Pareto efficiency, technical efficiency, allocation efficiency, exchange efficiency, and X-efficiency, but the essence is the same. Efficiency of outputs or profit maximization is inapplicable for evaluating multi-output production units. Because insurance company outputs are diversified this paper chose cost efficiency to examine corporate governance effectiveness. Yao et al. (2007) also selected technical efficiency and focused on cost minimization to study the efficiency of insurance companies. Frontier analysis is widely used to determine technical efficiency. It includes parametric analysis, such as DEA, and non-parametric analysis, such as SFA. SFA is more appropriate for efficiency studies in transition economies where measurement errors and uncertain economic environments are more likely to prevail (Fries and Taci, 2004 [40]; Sathye, 2003 [41]). Therefore, this paper selected SFA to examine the relationship between insurance corporate governance and a series of enterprise outputs to calculate the corporate governance effectiveness.

This study improves the model proposed by Battese and Coelli in 1995 [20].

Consider the stochastic frontier production function,

(1) $$Y_{\mathrm{it}}=\exp\left(\alpha_{it}\beta +V_{it}-U_{it}\right)$$

where $Y_{\mathrm{it}}$ denotes the production; $\alpha_{it}$ is the value of inputs functions and other explanatory variables; β is a (k × 1) vector of unknown parameters to be estimated; *I* denotes the *i*-th firm and *t* denotes the *t*-th year in the sample. $V_{it}$ is assumed to be iid*N* (0, $\sigma_{v}^{2}$) random errors, independently distributed of the $U_{it}$. $U_{it}$ denotes on-negative random variables associated with technical inefficiency of production. It is also assumed to be independently distributed, obtained by truncation (at zero) of the normal distribution with mean, $z_{it}\delta$, and variance, $\sigma_{}^{2}$. $z_{it}$ is a (1 × m) vector of explanatory variables associated with technical inefficiency of production and δ is an (m × 1) vector of unknown coefficients.

The technical inefficiency effect $U_{it}$ in model 1 could be specified using Equation (2),

(2) $$U_{\mathrm{it}}=z_{it}\delta +M_{it}$$

where the random variable $M_{it}$ is defined by the truncation of the normal distribution with zero mean and variance $\sigma_{}^{2}$.

Following the studies of Liu et al., (2007) [42], this paper chooses Cobb-Douglas production function as the stochastic frontier production function. It is estimated as

$$\ln\mathrm{cost}=\beta_{0}+\alpha \ln y+\beta_{1}\ln C_{1}+\beta_{2}\ln C_{2}+\beta_{3}\ln C_{3}+\varepsilon_{1}$$

(3) $$\ln\left({\mathrm{cost}}_{it}/C_{3}{}_{it}\right)=\beta_{0}+\alpha \ln y_{it}+\beta_{1}\ln(C_{1}{}_{it}/C_{3}{}_{it})+\beta_{2}\ln(C_{2}{}_{it}/C_{3}{}_{it})+\left(v_{it}+u_{it}\right)$$

where ln denotes the natural logarithm. 

The technical inefficiency effects are assumed to be defined by

$$u_{it}=\delta_{0}+\delta_{1}CG_{INDEX}+m_{it}$$

where y is the total value of the output, representing the incomes of insurance companies in business, profitability, and risk control; $C_{1}$, $C_{2}$, and $C_{3}$ are the investments of the insurance company in labor, capital, and material, respectively; and $CG_{INDEX}$ is the corporate governance variable obtained by factor analysis.

Battese and Coelli (1995) introduce exogenous factors into inefficiency function [20]. This approach examines the influence of corporate governance on cost efficiency and calculates the cost efficiency considering the corporate governance index. This is consistent with the definition in this paper. Insurance corporate governance is to weigh the difference of cost efficiency with consideration of the corporate governance indicator, given the exogenous environment.

## 4. Research Design

### 4.1. Variable Definitions

#### 4.1.1. Dependent Variables

In general, insurance outputs can be measured by premium revenues and profit. To examine the impact of corporate governance on risk control, this study also chose the solvency margin as an output variable. Therefore, according to the definition of production efficiency, dependent variables include income from premium revenues and investment income, profit, and solvency margin as output variables. This paper further calculated the insurance corporate governance effectiveness, including the business effectiveness, profitability effectiveness, and risk control effectiveness.

#### 4.1.2. Independent Variables

Defining the inputs and outputs of insurance companies is an important issue of this study. Following Cummins and Santomero (1999) [43], this paper argues that insurer inputs include commission charge, brokerage charge, and administrative expense. Following the same literature, production input factors include labor, capital, and materials. This paper chooses the labor input (calculated as commission expenses plus brokerage charge divided by its premiums revenues), capital input (equity divided by its total assets), and materials input (operating expenses divided by its total assets). Unlike industry companies, this study does not select the staff wages because insurance companies expand business mainly through the insurance agents and intermediary institutions, so brokerage charges better reflect the actual labor costs.

This study designed a set of governance evaluation indexes for joint-stock insurance companies that reflects their characteristics. The index system is based on the special institutional background of China’s insurance companies, and refers to the evaluation index in the Insurance Company Governance Report of the CIRC. Consistent with prior research, these key governance variables include eight primary indicators—such as shareholder governance, board of directors, board of supervisors, managerial governance, new three sessions, directors supervisor, internal audit, and external supervision—as well as 52 secondary indicators, and 94 tertiary indicators. For positive indicators, ‘yes’ was assigned a value of 1, and ‘no’ responses were assigned a value of 0; and for reverse indicators, ‘yes’ was assigned a value of 0, and ‘no’ responses were assigned a value of 1. Then the study summed the score to obtain the results of the evaluation of insurance company governance. This paper constructed an internal governance index (CG_INDEX_) by factor analysis for studying the overall effect of corporate governance. This study determined the index weights according to the variance contribution rate, while referring to the evaluation system used by the listed banks’ corporate governance database from the China Center for Economic Research (CCER).

This study differentiated sample insurance companies, according to several important influencing factors, to distinguish the governance effectiveness of different insurance companies. The first influencing factor was whether the company is controlled by the government or its agent. The study considers that insurers are considered to be controlled by the government if the largest shareholder is the finance bureau, a government investment corporation, or a local state-owned enterprise. The second factor chosen was whether the company was listed on the stock exchanges. Listed companies must adhere to more stringent regulations, so the corporate governance effectiveness may be stronger. The last factor was whether the insurer was primarily a life or property insurer. The insurance term for life insurance is much longer than that of property insurance. Therefore, corporate governance of life insurance ought to be more effective. The regulatory requirements for life insurance by the CIRC are more stringent.

### 4.2. Data 

This study collected data from Chinese joint-stock insurance companies in 2010 and 2011. In 2010, there were 64 joint-stock insurance companies in China. Then it distributed 64 questionnaires and received 59 in response. Excluding the questionnaires with incomplete or abnormal data, this study collected 43 valid questionnaires for that year. There were 69 joint-stock insurance companies in 2011, and it collected 42 pieces of valid information by the same procedure. The sample contains observations of all major Chinese joint-stock insurance companies, accounting for more than 90% of total insurer assets. The questionnaire, which was completed by the office of the board of directors of each insurance company, was issued in cooperation with the CIRC. This study was authorized to use this information for academic research. Other core data, such as financial data and basic information about these companies, were obtained from the China Insurance Statistics Yearbook 2011–2012. To avoid the endogeneity in empirical research, the paper chose the output data with a time lag of one year.

## 5. Empirical Results

### 5.1. Reliability Analysis and Validity Analysis

#### 5.1.1. Reliability Analysis 

The study interviewed the CIRC supervisors and the directors of 12 insurance companies when designing the questionnaire, and analyzed the internal consistency by Cronbachα coefficient. The improved results are shown in Table 1. Cronbachα coefficients of all dimensions are greater than 0.7, which indicates the higher reliability of the questionnaire and conforms to the research standard.

#### 5.1.2. Structural Validity Factor Analysis

This study used the exploratory factor analysis method to test the construct validity. The KMO value of this research was 0.771, which is greater than 0.7. The significance probability of the sphericity test of Bartlett in the table was 0.000, indicating that factor analysis could be performed.

### 5.2. Descriptive Analysis

Table 2 reports the summary statistics of our main variables. The CG_INDEX_ is calculated by factor analysis, and its value does not have practical significance, but it is comparative between sample companies. While the smallest figure is 0.241, the highest is 0.973. The large differences in the proxies for insurers’ governance behavior in our sample facilitate our analysis. The mean income is 20,206.513 million Yuan, ranging from 5.221 million to 388,791.599 million, while the mean profit is 1310.748 million Yuan, ranging from −932.110 million to 41,745.061 million. This reflects a huge discrepancy between Chinese insurance companies’ market share and profitability. The large range and standard deviation of solvency margin also verify this finding. Significantly, the solvency margin of the China Insurance Company (CIC) in 2011 is only −16,509.142 million Yuan. 

### 5.3. Results of Stochastic Frontier Model

Maximum-likelihood estimates of the stochastic frontier are reported in Table 3. This paper examines corporate governance effectiveness from three aspects, namely, business, profitability and risk control. The dependent variables in the three models are income, profit, and solvency margin, respectively. The estimated *r* in models 1 and 3 are more than 0.820 and significant at the 1% level. The LR tests of one-sided error are 36.430 and 19.729, greater than 9.500. They are significant at the 1% level according to Kodde and Palm (1986) [44]. 

The results prove the existence of inefficiency in insurance company cost effective models 1 and 3. The *δ*_1_ in inefficiency function reflects the impact of corporate governance on cost efficiency. The corporate governance could improve the cost efficiency if the *δ*_1_ is negative. The *δ*_1_ are significant at the 1% level in model 1 and at the 10% in model 3. These results verify the Hypotheses 1 and 3. Meanwhile, the *r* of model 2 is only 0.710, and the *δ*_1_ is not significant in model 2. 

The results show that corporate governance effectiveness is different in all three investigated areas. Corporate governance effectiveness is more obvious in improving business and risk control abilities, while its impact on profitability has not been confirmed. This study considers that this result is because corporate governance is an institutional guarantee, so its effectiveness is reflected mostly in risk control. China’s insurance companies are still in the stage of extensive growth. Their rapid expansion in market share could result in huge premium income, but the dangers of cost and risk control are still unknown. In contrast to these two types of effectiveness, there are two reasons why the profitability effectiveness of corporate governance is not evident. On one hand, profitability is a consequence of corporate marketing activities, and is more closely related to the management of insurance companies. Moreover, corporate governance focuses on top-level design and has little direct effect on enhancing profitability. On the other hand, the current focus of China’s insurance companies is still on occupying the market. Therefore, given this expansion, the profitability is weak in this stage.

### 5.4. Insurance Corporate Governance Effectiveness

This study calculate the cost effectiveness (EFF value), considering corporate governance according to the model constructed above. The higher EFF value indicates the lower cost efficiency. It can also calculate the EFF value without reference to the corporate governance index. The difference between the two EFF values can reflect corporate governance effectiveness. Its summary statistics are shown in Table 4. 

The mean of risk control effectiveness is larger than that of business effectiveness, which is consistent with the stochastic frontier results. This shows that the effect of insurance corporate governance on risk control is more obvious. This phenomenon may be related to the current business model of the insurance companies. It is difficult for corporate governance to affect business and market behavior in pursuit of market share and rapid expansion. A larger standard deviation of risk control effectiveness reflects a huge discrepancy among the influence of corporate governance on risk control of Chinese insurance companies. 

The business effectiveness of non-state insurers is better than that of state-owned insurers, while their risk control effectiveness is the opposite. The results show that corporate governance plays a more important role in risk control for state-owned insurers. A number of reasons account for this result: first, state-owned insurance companies functioned as government agents in the past and still have to maintain the stability of the insurance industry; second, in highly concentrated markets, profitability is easy to attain for large state-owned enterprises; finally, the evaluation and assessment of state-owned enterprises in China places greater importance to risk control. Therefore, the executives of state-owned insurers usually are interested in gaining merit and in avoiding risks.

Listed insurers outperformed unlisted insurers in both business and risk control effectiveness. Empirical studies have generally revealed the positive association between enterprise performance and listing on exchanges in developing countries and transition economies. One possible reason for this is that IPO facilitates the adoption of market-oriented disciplines and good corporate governance. According to our definition, the benefit of corporate governance is the reduction of the principal-agent and capital costs. Listed insurers have to alter their corporate governance to solve the principal-agent problem and improve efficiency for compliance and attracting investors. Exposing the value of corporate governance for non-listed insurers over a short time frame is difficult. Therefore, non-listed insurers lack the motivation and incentive to improve corporate governance. 

In the last comparison, life insurers perform better than property insurers in terms of effectiveness. The policy period for life insurance is significantly longer than that of property insurance, making the contract more complex. Long-term interests of policy holders must be guaranteed after contracting; thus, the management of life insurance companies is more important so the company will be able to meet its contractual obligations. Appropriate corporate governance is more valuable for life insurers from both the regulatory and insurance company viewpoint.

## 6. Conclusions

This study defined the concept of corporate governance effectiveness, and put forward a reasonable measurement method. Then it applied the method to a sample of China’s insurance companies. The results show that the effectiveness is as varied as the three aspects of business ability, profitability, and risk control. Insurance corporate governance could improve a company’s business ability, but the significance was weaker because this study could not find significant evidence to prove the influence of insurance corporate governance on profitability. The function of insurance corporate governance in risk control was the most obvious as insurers pay more attention to business results than to governance.

State-owned insurers tended to outperform non-state firms in corporate governance effectiveness, reflecting the goal of state-owned insurers of avoiding individual and industrial risks. Corporate governance effectiveness of listed insurance companies is significantly higher than that of non-listed companies, associated with a more complete governance structure of listed companies, and proves that the corporate governance allows the ability to work with more complex principal-agent problems. This paper also found that life insurance corporate governance is more effective, showing that corporate governance plays a more prominent role in life insurance companies, especially in controlling long-term risks, consistent with the stricter governance requirements by the regulatory authorities.

This study contributes to the literature in several ways. First, it suggests that corporate governance enhances companies’ efficiency by affecting the costs of input factors and resource allocation efficiency. This paper defined corporate governance effectiveness from the perspective of efficiency as the efficiency improvement as a result of corporate governance. Then, the study divided the insurance corporate governance effectiveness into three aspects, namely business effectiveness, profitability effectiveness, and risk control effectiveness. This practice not only enriched the corporate governance theory but also clarified the function of corporate governance in an enterprise, combined with the new institutional economics theory. Secondly, it examined the insurance corporate governance effectiveness with a non-efficiency function, based on the model proposed by Battese and Coelli in 1995. The results verified the existence of corporate governance effectiveness and calculated a value, which quantified the effectiveness. The definition and measurement of corporate governance effectiveness in this paper are consistent and creative. The contributions provide a feasible method for the theoretical and empirical study of corporate governance effectiveness. This method can be widely applied to the study of corporate governance in various countries and industries. Third, the study examined the effectiveness of insurer governance from the unique data obtained from regulators. The results are conducive to obtaining a comprehensive understanding of insurance corporate governance effectiveness but also for the establishment of empirical research on the governance of China’s insurance industry.

This study also provides important implications for policy makers in transition economies. China’s insurance regulatory authorities can reform the contents and approach of regulation in accordance with our results, and further strengthen the basic safeguard of corporate governance to guide enterprises to strengthen the effectiveness of corporate governance in terms of voluntary profitability. Better regulation could assist in the transition from mandatory regulation to voluntary regulation by providing a reference for external regulation in emerging economies. 

Although this study obtained the most comprehensive and available data, the coverage of the data period is still relatively limited, which is reflected in the results and conclusions in this paper. The effect of current insurance company governance is more reflected in the insurance company itself. Since 2016, China’s insurance companies have played a more active role in the capital market. Insurers have acquired other industrial companies, and transformed from being governed to the governor. How a company’s own governance mechanism affects mergers and acquisitions is yet to be examined. The spillover effect of corporate governance is one of the recommended directions for future research.

## Figures and Tables

**Table 1:** Cronbachα coefficients.

	Cronbachα Coefficient	Number of Items
Shareholder Governance	0.879	12
Board of Directors	0.821	12
Board of Supervisors	0.768	12
Managerial Governance	0.799	12
New Three Sessions	0.836	12
Directors Supervisor	0.901	12
Internal Audit	0.847	12
External Supervision	0.766	12

**Table 2:** Summary statistics of the main variables (million Yuan).

	Observation	Mean	Standard Deviation	Min	Max
Income	85	20,206.513	59,713.162	5.221	388,791.599
Profit	85	1310.748	6376.689	−932.110	41,745.061
Solvency Margin	85	2613.164	14,906.798	−16,509.142	98,660.012
Brokerage Charge	85	1554.415	4363.087	0.073	27,256.009
Administrative Expense	85	2029.069	4257.873	4.645	20,917.000
Premium Revenues	85	18,159.420	50,542.310	0.555	318,229.000
CG_INDEX_	85	0.849	0.145	0.241	0.973
Size (ln)	85	3.871	0.752	2.597	6.158

Note: The variables related to corporate insurance were calculated from questionnaires completed through the insurance regulatory authority. Financial and basic information variables were calculated from the China Insurance Statistics Yearbook 2009–2011.

**Table 3:** The influence of insurance corporate governance on cost efficiency.

	(1)	(2)	(3)
	Income	Profit	Solvency Margin
Cost function			
*β*_0_	5.882 ***	4.460 ***	7.330 ***
	13.181	7.997	8.164
*Α*	0.004 ***	0.496 ***	−0.181 *
	4.298	4.739	−1.720
*β*_1_	0.124 ***	0.176 ***	0.129 ***
	2.791	7.174	2.695
*β*_2_	−0.136 ***	−0.185 ***	−0.114 ***
	−5.032	−16.443	−3.188
Inefficiency Function			
*δ*_0_	−19.616	−2.947 **	−1.271
	−0.152	−2.336	−0.299
*δ*_1_	−0.106 *	−3.065	−1.669 ***
	−1.713	−0.866	−3.892
Coefficient Variation			
*σ*^2^	15.559	1.990 **	4.941
	0.188	2.296	1.057
*γ*	0.942 ***	0.710 **	0.900 ***
	3.177	2.448	9.046
*LR*	36.430	28.378	19.729

Note: The sample included 43 Chinese insurance companies in 2009 and 42 companies in 2010. The numbers in the test-of-difference columns denote the *t* value; ***, **, and * denote significance at the 1%, 5%, and 10% level, respectively.

**Table 4:** Summary statistics of total insurers corporate governance effectiveness.

	Obs.	Mean	Std. Dev.	Min	Max
Business Effectiveness	85	0.879	0.629	−1.211	2.318
Risk Control Effectiveness	85	1.653	5.675	−14.216	6.791

Note: The results were calculated by Frontier 4.1 (Centre for Efficiency and Productivity Analysis, Brisbane, Australia). Because there is no evidence for Profitability Effectiveness, we only reported the Business Effectiveness and Risk Control Effectiveness here.
